# Therapeutic and prophylactic deletion of IL‐4Ra‐signaling ameliorates established ovalbumin induced allergic asthma

**DOI:** 10.1111/all.14137

**Published:** 2020-01-30

**Authors:** Jermaine Khumalo, Frank Kirstein, Martyna Scibiorek, Sabelo Hadebe, Frank Brombacher

**Affiliations:** ^1^ Division of Immunology, and South African Medical Research Council (SAMRC) Immunology of Infectious Diseases Department of Pathology Faculty of Health Sciences University of Cape Town Cape Town South Africa; ^2^ Division of Immunology Health Science Faculty International Centre for Genetic Engineering and Biotechnology (ICGEB) and Institute of Infectious Diseases and Molecular Medicine (IDM) University of Cape Town Cape Town South Africa; ^3^ Faculty of Health Sciences Wellcome Centre for Infectious Diseases Research in Africa (CIDRI‐Africa) Institute of Infectious Diseases and Molecular Medicine (IDM) University of Cape Town Cape Town South Africa

**Keywords:** IL‐4Rα, prophylactic, tamoxifen, T_H_2 type, therapeutic

## Abstract

**Background:**

Allergic asthma is a chronic inflammatory airway disease driven predominantly by a T_H_2 immune response to environmental allergens. IL‐4Rα‐signaling is essential for driving T_H_2‐type immunity to allergens. Anti‐T_H_2 therapies have the potential to effectively reduce airway obstruction and inflammation in allergic asthma.

**Objective:**

We investigated potential therapeutic effects of selective inhibition of this pathway in mice with established allergic airway disease. We further investigated whether IL‐4Rα disruption in systemically sensitized mice can prevent the onset of the disease.

**Methods:**

We used Rosa^creERT2^IL‐4Rα^−/lox^ mice, a tamoxifen (TAM)‐inducible IL‐4Rα knockdown model to investigate the role of IL‐4/IL‐13 signaling prior to the onset of the disease and during the effector phase in the ovalbumin‐induced allergic airway disease.

**Results:**

Inducible deletion of IL‐4Rα demonstrated therapeutic effects, on established allergic airway disease, and prevented the development of ovalbumin‐induced airway hyperreactivity, eosinophilia, and goblet cell metaplasia in allergen‐sensitized mice. Interestingly, IL‐4Rα knockdown after allergic sensitization did not induce T_H_17, a neutrophilic inflammatory response as observed in global IL‐4Rα‐deficient mice after intranasal allergen challenge.

**Conclusion:**

Abrogation of IL‐4Rα signaling after allergic sensitization would have significant therapeutic benefit for T_H_2‐type allergic asthma.

## Graphical abstract

Interleukin 4 receptor alpha (IL‐4Rα) is central in the initiation and maintenance of the T_H_2 allergic airway disease. Temporal genetic deletion of the IL‐4Rα after sensitization reduced T_H_2 disease by abrogating effector mechanisms such as CD11b^+^ migratory DCs, T_H_2‐associated cytokines, eosinophilia, mucus production, and AHR. Temporal genetic deletion of the IL‐4Rα in an established disease reduced CD11b^+^ migratory DCs, eosinophilia, IgE, mucus production, AHR, but not T_H_2‐associated cytokines. Global deletion of the IL‐4Rα induces CD103^+^ DCs, neutrophilia, and TH17 cytokines, underscoring the importance of temporal genetic deletion. Abbreviations: AHR, airway hyperresponsiveness; IL‐4Ra, interleukin 4 receptor alpha; RORt, RAR‐related orphan receptor gamma; STAT6, signal transducer and activator of transcription 6

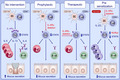

## 1 INTRODUCTION

Airway hyperresponsiveness (AHR), pulmonary inflammation, eosinophilia, and mucus hyperplasia are hallmarks of allergic asthma. Indeed, chronic disease is driven predominantly by T_H_2 immune responses with pathology largely caused by IL‐4 and IL‐13 signaling, which share a common pleiotropic receptor subunit, interleukin 4 receptor alpha (IL‐4Rα).^1^, ^2^, ^3^, ^4^ The IL‐4/IL‐13 axis has been a target for allergic asthma treatment; however, anti‐T_H_2‐based therapies for asthma have seen limited success in clinical trials.^4^, ^5^, ^6^ The therapy conundrum is further compounded by phenotypic heterogeneity among asthma cases, limited treatment options for steroid‐resistant cases, and possible non‐T_H_2 inflammatory mechanisms involved in asthma pathogenesis.^7^, ^8^, ^9^, ^10^, ^11^ However, targeting T_H_2‐type inflammation still remains a promising therapeutic approach for a large proportion of carefully stratified asthma patients.^9^

Key in the IL‐4/IL‐13 axis is the IL‐4Rα signaling which has been identified as a potential target for asthma therapies.^12^, ^13^, ^14^ Deficiency of IL‐4Rα in allergen‐sensitized mice shows reduced allergen‐induced symptoms such as AHR, eosinophilia, and mucus hyperplasia in vivo.^15^, ^16^, ^17^ We and others have also illustrated various cell‐specific roles of IL‐4Rα signaling during the development of allergic disease pathology.^11^, ^18^, ^19^, ^20^, ^21^ However, limited successful anti‐T_H_2‐based treatments for asthma calls for better understanding of the mechanisms involved in successful therapy. Hence, we investigated in vivo requirements of IL‐4Rα signaling after allergic sensitization prior to the onset of disease as well as after T_H_2 pulmonary allergic airway lung inflammation. We hypothesized that temporal significance of IL‐4Rα signaling might highlight its prophylactic and therapeutic relevance, especially as most patients are diagnosed only after established exacerbations.^22^, ^23^ Resurgence of side effects such as T_H_17 responses has been a concern for anti‐T_H_2 targeted therapies.^4^, ^5^, ^6^, ^24^, ^25^, ^26^, ^27^, ^28^

We developed a TAM‐induced, conditional IL‐4Rα knockdown mouse model (Rosa^creERT2^IL‐4Rα^−/lox^) and sought to investigate the temporal role of IL‐4Rα receptor signaling during the effector phase (therapeutic) and priming/sensitization phase (prophylactic) of allergic asthma. Rosa^creERT2^IL‐4Rα^−/lox^, IL‐4Rα^−/lox^, and IL‐4Rα^−/−^ mice were sensitized with OVA/alum complex intraperitoneally and challenged intranasally with OVA to induce allergic airway disease. Temporal deletion of IL‐4Rα chain after sensitization phase prevented disease development, whereas temporal deletion during the effector phase reduced most disease parameters, such as AHR, eosinophilia, as well as goblet cell metaplasia. Unexpectedly, temporal deletion of IL‐4Rα signaling in both prophylactic and therapeutic models did not develop T_H_17‐type and neutrophilic airway inflammation, a phenotype completely different to global deletion of IL‐4Rα. We, thus, conclude that abrogation of IL‐4Rα signaling after allergic sensitization would have significant therapeutic benefit for T_H_2 type asthma without inducing potentially detrimental T_H_17 responses.

## 2 METHODS

### 2.1 Mice

We generated an inducible IL‐4Rα deletion mouse*,* Rosa^creERT2^IL‐4Rα^−/lox^ 29 on a BALB/c background by intercrossing transgenic Rosa^creERT2^IL‐4Rα^−/lox ^C57BL/6 mice^29^ with IL‐4Rα*^lox/lox^* BALB/c mice (Figure S1). IL‐4Rα^−/lox^ 30and IL‐4Rα^−/−^ 31mice on a BALB/c background were used as control animals. Eight‐ to twelve‐week‐old mice were used for the experiments and housed in independently ventilated cages under specific pathogen‐free conditions in the University of Cape Town Animal Facility. Animal procedures were approved by the University of Cape Town Animal Ethics Committee (reference number, 015/009).

### 2.2 Models of allergic airway disease

#### 2.2.1 Prophylactic model: Postsensitization IL‐4Rα knockdown in ovalbumin‐induced allergic airway disease

Mice were sensitized intraperitoneally with (50 µg in 200 µL) of ovalbumin (OVA) adsorbed to 0.65% alum (Sigma‐Aldrich) on days 0, 7, 14.^32^ The TAM and oil treatment was done by oral gavage of 100 µL of 2.5 mg/d tamoxifen solubilized in vegetable oil (OIL) or 100 µL of vegetable oil, respectively, on days 15, 16, 17, 18. On days 23, 24, 25, mice were intranasally challenged with 100 µg of OVA under anesthesia with anaesthetized with ketamine (Anaket‐V; Centaur Labs) and xylazine (Rompun; Bayer) as previously described.^32^ AHR was measured on day 26. After the procedure, mice were euthanized with halothane and tissue samples collected for analysis.

#### 2.2.2 Therapeutic model: Posteffector phase IL‐4Rα knockout in ovalbumin‐induced allergic airway disease

Mice were sensitized intraperitoneally with (50 µg in 200 µL) of OVA/alum on days 0, 7, 14. On days 23, 24, 25, mice were intranasally challenged with 100 µg of OVA under anesthesia. TAM and OIL treatment was done on days 26, 27, 28, 29. Mice were intranasally challenged again on days 34, 35, 36, and AHR was measured on day 37. After the procedure, mice were euthanized and tissue samples were collected for analysis.

### 2.3 Lung function measurements

Airway resistance and elastance of the whole respiratory system (airways, lung chest wall) after intranasal challenge was determined by forced oscillation measurements as described previously^32^ with the Flexivent system (SCIREQ) by using the single compartment (“snapshot”) perturbation. Measurements were carried out on mice with increasing doses of acetyl‐ß‐methylcholine (methacholine, Sigma‐Aldrich) treatment. Differences in the dose‐response curves were analyzed by repeated‐measures ANOVA with the Bonferroni post‐test. Only mice with acceptable measurements for all doses (coefficient of determination > 0.90) were included in the analysis.

### 2.4 Analysis of cell populations by flow cytometry

Bronchoalveolar lavage (BAL) cells were obtained as previously described.^19^ Single‐cell suspensions were prepared from lymph nodes in Iscove's modified Dulbecco's medium (IMDM) (Gibco) by passing them through 40‐µm filter. To obtain single‐cell suspensions from lung tissues, a left lobe lung was digested for 1 hour at 37°C in RPMI (Gibco, Paisley, United Kingdom) containing 13 mg/mL DNase I (Roche) and 50 U/mL collagenase IV (Gibco) and passed through 70‐µm filter. Single cells were then blocked with 24G2 for 30 minutes at 4°C, followed by surface staining with fluorophore‐conjugated antibodies for 30 minutes at 4°C in the dark.

Antibodies used in these experiments included phycoerythrobilin (PE)‐conjugated anti‐Siglec‐F (clone, E50‐2440), and anti‐CD124 (IL‐4Rα, clone, M‐1), FITC‐conjugated anti‐Gr‐1 (clone, RB6‐8C5), PerCPCy5.5‐conjugated anti‐Ly6C (clone, AL‐21), ‐CD45.1 (clone, A20), Allophycocyanin (APC)‐conjugated anti‐CD11c (clone, HL3), V450‐conjugated anti‐CD11b (clone, M1/70), AlexaFlour 700‐conjugated anti‐CD3ε (clone, 145‐2C11), V500‐anti‐CD4 (clone, RM4‐5) and anti‐B220 (clone, RA3‐6B2), APC‐Cy7‐conjugated anti‐CD19 (clone, 1D3), and anti‐CD8 (clone, 53‐6.7) were purchased from BD Biosciences, PE‐Cy7 anti‐F4/80 (clone, BM8), AlexaFlouro 700‐conjugated anti‐MHC II (clone, M5/114), and live/dead fixable yellow stain (Qdot605 dead cell exclusion dye) were purchased from eBiosciences. For intracellular cytokine staining, surface‐stained cell suspensions were fixed in 2% PFA, permeabilized with 0.5% saponin buffer, and stained with PE‐conjugated p‐STAT6 (clone, J71‐773.58.11 BD Biosciences). Cells were acquired using Fortessa (BD Biosciences), and data were analyzed with FlowJo version 10 software (TreeStar).

### 2.5 Histology and immunohistochemistry

Lungs were fixed in 4% formaldehyde/PBS and embedded in paraffin. Tissue sections were stained with periodic acid‐Schiff (PAS) for mucus secretion, hematoxylin and eosin (H&E) staining for inflammation. Image analysis was performed on NIS Elements (Nikon Instruments). Mucus quantification was carried out using the automated NIS Elements software by defining regions of interest (ROIs) which are the individual bronchioles on cut lung sections to be analyzed for mucus staining and using threshold quantification of the mucus stain in the specific ROIs NIS Elements (Nikon Instruments). Area of staining is defined as total area of mucus secretion per area of bronchiole epithelial lining. Lung sections from individual mice were assessed, and data from 3 experiments were pooled (n = 4‐6 mice per experiment).

### 2.6 Antibody and cytokine ELISAs

Antibody ELISAs were carried out as previously described 19 using 1 mg/mL OVA to coat for specific IgE and 0.5 mg/mL to coat for IgGs. For in vitro cytokine production analysis, single‐cell suspensions were prepared from mediastinal lymph nodes of OVA‐treated and littermate control mice.^19^ Cells (2 × 10^5^ cells, in 200 µL) were incubated for 5 days in IMDM/10% FCS (Delta Bioproducts) in 96‐well plates. Cells were either stimulated with OVA (50 µg/mL) or anti‐CD3 (10 µg/mL) and supernatants were collected after incubation period. Concentrations of IL‐2, IL‐4, IL‐5, IFN‐*γ*, (BD Biosciences), IL‐13 (R&D Systems), IL‐17, and IL‐23 (BioLegend) were measured using ELISA assays according to the manufacturer's protocol.

### 2.7 Statistical analysis

*P* values were calculated in GraphPad Prism 6 (GraphPad Software, Inc) by using nonparametric Mann‐Whitney Student's *t* test or two‐way ANOVA with Bonferroni's post‐test for multiple comparisons, and results are presented as SE of the mean. Differences were considered significant if *P* was <.05.

## 3 RESULTS

### 3.1 Characterization of IL‐4Rα expression in Rosa^creERT2^IL‐4Rα^−^^/lox^ mice

**Figure 1:**
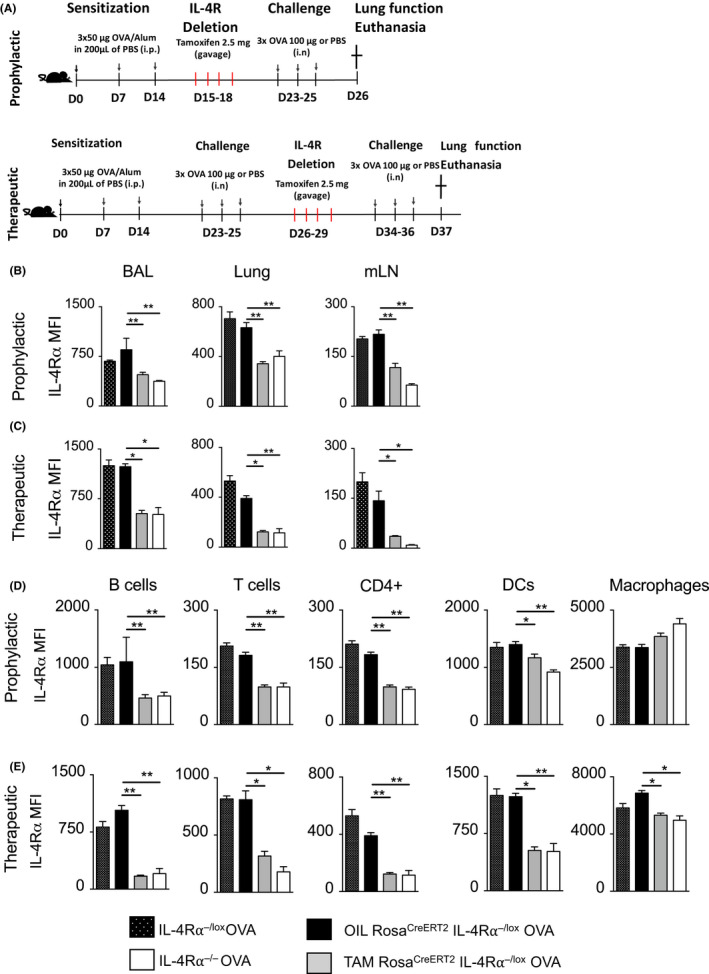
Prophylactic and therapeutic inducible deletion of IL‐4Rα in OVA‐induced allergic airway inflammation. A, Schematic diagram of IL‐4Rα deletion using the tamoxifen (TAM)‐inducible mouse model (ROSA^creERT2^IL‐4Rα^−/lox^ mice) in a prophylactic (top panel) and therapeutic model (bottom panel). Mice were fed TAM (2.5 mg) orally for 4 d either after sensitization (prophylactic) or after first challenge (therapeutic). B, IL‐4Rα expression in different organs including bronchoalveolar lavage fluid (BALF), lung, and mediastinal lymph nodes (mLNs) in the prophylactic model. C, IL‐4Rα expression in different organs including BALF, lung, and mLNs in the therapeutic model. D, IL‐4Rα expression in different lung cell types including B cells (CD19^+^MHCII^+^), T cells (CD3^+^ MHCII^−^CD11c^−^), CD4^+^ T cells (CD3^+^CD4^+^MHCII^−^CD11c^−^), dendritic cells (CD11c^high^SiglecF^low^ MHCII^hi^), and alveolar macrophages (CD11c^hi^SiglecF^hi^ MHCII^low^) in a prophylactic model. E, IL‐4Rα expression in different lung cell types including B cells (CD19^+^MHCII^+^), T cells (CD3^+^MHCII^−^CD11c^−^), CD4^+^ T cells (CD3^+^CD4^+^MHCII^−^CD11c^−^), dendritic cells (CD11c^high^ SiglecF^low^ MHCII^hi^), and alveolar macrophages (CD11c^hi^SiglecF^hi^ MHCII^low^) in a therapeutic model. Data shown mean ± SDs from 1 representative experiment of 3, n = 5. Significant differences to *IL‐4Ra* ^−/−^ OVA: *P < .05, **P < .01, ***P < .001

Inducible Rosa^creERT2^IL‐4Rα^−/lox^ mouse strain has been previously characterized on a C57BL/6 background strain by our laboratory and shown to have impaired expression of IL‐4Rα on lung and lymph node tissue upon TAM treatment.^33^ Here, we characterized IL‐4Rα expression in Rosa^creERT2^IL‐4Rα^−/lox^ mouse strain on the BALB/c background under OVA‐induced allergic asthma. The tamoxifen‐induced deletion of IL‐4Rα lasts for more than 16 weeks after 4 days of tamoxifen instillation by oral gavage with no homeostatic imbalance.^33^ We induced deletion of IL‐4Rα in two models, prophylactic (where mice were fed TAM orally after sensitization before acute challenge with OVA) and therapeutically (where mice were fed TAM orally after sensitization and acute challenge with OVA) (Figure 1A). Efficient knockdown of IL‐4Rα in both lung and mediastinal lymph node (mLN) tissue after TAM treatment compared to vehicle‐treated mice was observed (Figure 1B,C). We further evaluated cell type–specific deletion of IL‐4Rα and found significantly reduced expression in inflammatory cells such as CD4^+^ T cells, B cells, and dendritic cells (DCs), but not macrophages in lung tissue of TAM‐treated mice compared to the OIL‐treated littermate controls (Figure 1D,E). Expression levels of IL‐4Rα after TAM knockdown were similar to those observed in IL‐4Rα global knockout mice (Figure 1B‐E). We therefore concluded that IL‐4Rα was efficiently knocked down upon treatment with TAM.

### 3.2 Knockdown of the IL‐4Rα signaling prophylactically prevented the development of AHR and airway inflammation

**Figure 2:**
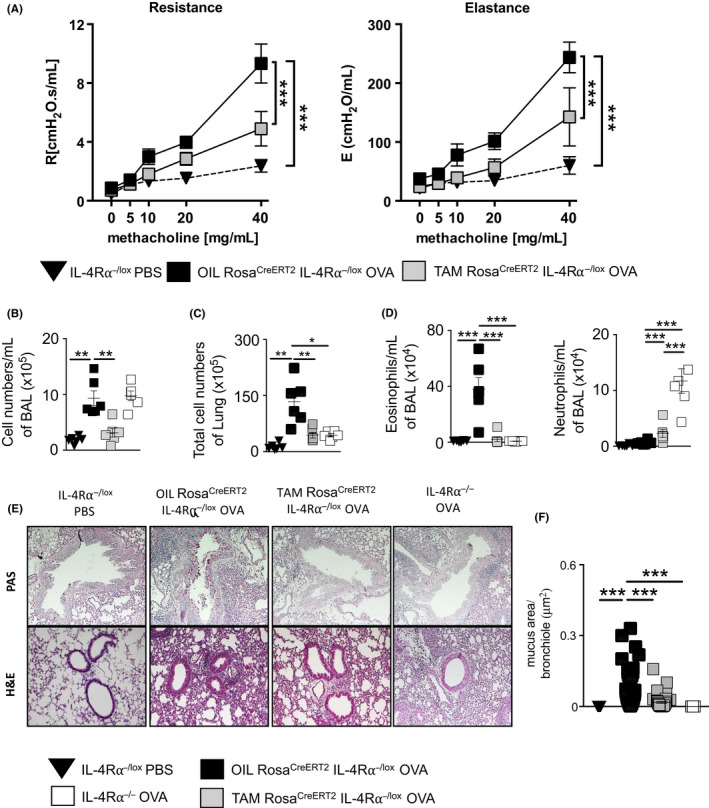
Prophylactic deletion of the IL‐4Rα restores normal lung function and reduces allergic airway inflammation. Mice (ROSA^cre ERT2^IL‐4Rα^−/lox^) were sensitized with ovalbumin (OVA)/Alum on days 0, 7, and 14 and fed TAM or OIL on days 15 to 18 and challenged with OVA on days 23‐25. Analysis was done on day 26. A, Airway resistance and airway elastance were measured with increasing doses of methacholine (0‐40 mg/mL). B, Total bronchoalveolar lavage fluid cells. C, Total lung cells. D, Total number of neutrophils (CD11c^low^CD11b^high^Ly6G^high^) and eosinophils (CD11c^low^ CD11b^high^ Ly6G^low^ SiglecF^hi^) in the BALF were stained and analyzed by flow cytometry. E, Histology analyses of lung sections (magnification ×200), stained with hematoxylin and eosin (H&E) (bottom) and periodic acid‐Schiff (PAS) (top). F, Automated quantification of the area (μm^2^) of mucus staining per analyzed bronchiole epithelial lining was carried out using NIS elements imaging software. Shown is mean ± SDs from one representative of 3 independent experiments (n = 5‐8). Significant differences are described as: *P < .05, **P < .01, ***P < .001

Mice deficient in IL‐4Rα are protected from OVA‐induced allergic airway disease.^1^ Here, we explored the temporal requirement of this signaling pathway postsensitization with OVA/alum. We assessed AHR after OVA challenge and found TAM‐Rosa^creERT2^IL‐4Rα^−/lox^ mice to have significantly reduced airway resistance and elastance compared to the OIL‐Rosa^creERT2^IL‐4Rα^−/lox^ littermate controls (Figure 2A). We also observed significantly increased resistance and elastance in OVA‐challenged and OIL‐Rosa^creERT2^IL‐4Rα^−/lox^ when compared to PBS‐challenged control mice. This demonstrated that deletion of IL‐4Rα after sensitization prevented the development of AHR following allergen challenge.

We measured total numbers of infiltrating cells, eosinophils, and neutrophils in the BAL fluid and lung tissue and found that total cells and eosinophils were reduced in TAM‐Rosa^creERT2^IL‐4Rα^−/lox^ mice compared to OIL‐Rosa^creERT2^IL‐4Rα^−/lox^ mice (Figure 2B‐D). We observed increased neutrophil infiltration in IL‐4Rα^−/−^ mice, which was absent in TAM‐Rosa^creERT2^IL‐4Rα^−/lox^ mice (Figure 2D). Furthermore, we observed reduced mucus hypersecretion (Figure2E,F) and lung inflammation (Figure 2E) in TAM‐Rosa^creERT2^IL‐4Rα^−/lox^ mice compared to OIL‐Rosa^creERT2^IL‐4Rα^−/lox^ mice. Taken together, these data demonstrate that IL‐4Rα‐dependent signaling postsensitization is important in regulating airway inflammation and that IL‐4Rα‐dependent signaling prior to sensitization may contribute to airway neutrophilia.

### 3.3 IL‐4Rα signaling is necessary for inducing a T_H_2 allergic airway immune response in a prophylactic model

**Figure 3:**
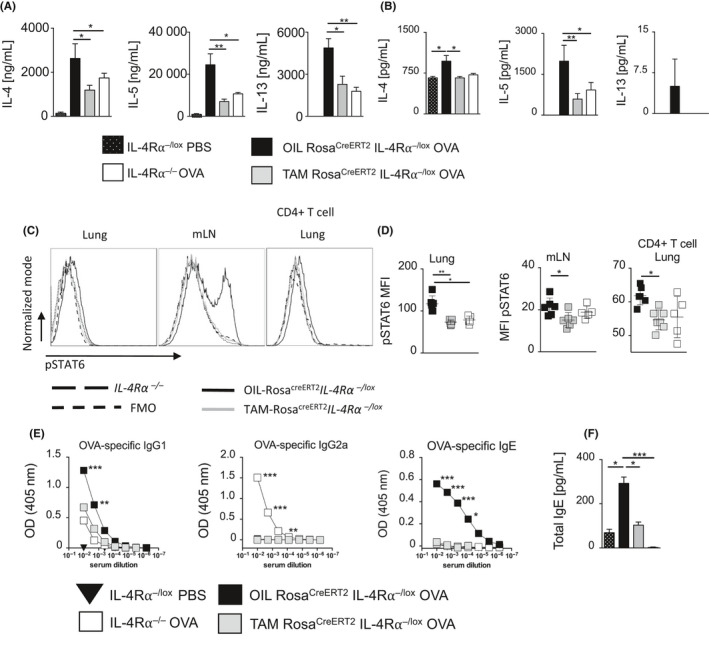
Prophylactic deletion of the IL‐4Rα impairs the induction of a T_H_2 cell–mediated cytokine and humoral response in allergic airway inflammation. T_H_2‐associated cytokine production in prophylactic ovalbumin (OVA) induced allergic asthma (as described in Figure 1, A) was measured in ex vivo restimulated mediastinal lymph nodes (mLN) by ELISA. A, anti‐CD3 stimulated and B, OVA stimulated). C and D, Histogram showing impairment of pSTAT6 expression in OVA‐challenged mLN, lung tissue, and lung CD4^+^ T cells of ROSA^cre ERT2^IL‐4Rα^−/lox^ mice after induced gene deletion was evaluated by flow cytometry and represented as MFI expression histograms of pSTAT6 in the prophylactic model. Data representative mean ± SDs from one of two experiments (n = 5‐7). MFI, median fluorescence intensity; p‐STAT6, phosphorylated signal transducer and activator of transcription 6; FMO, fluorescence “minus” one. E, Antibody production in prophylactic depletion model; OVA‐specific IgG_1_, IgG_2a,_ and IgE; F, Total IgE. Data show mean ± SDs from 1 representative experiment of 3 independent experiments carried out (n = 5). Significant differences are described as: *P < .05, **P < .01, ***P < .001

We measured T_H_2 cytokines after stimulation of mLNs with anti‐CD3 (Figure 3A) or OVA antigen (Figure 3B) in prophylactic model. Temporal deletion of IL‐4Rα in a prophylactic model significantly reduced IL‐4, IL‐5, and IL‐13 production in TAM‐Rosa^creERT2^IL‐4Rα^−/lox^ mice compared to OIL‐Rosa^creERT2^IL‐4Rα^−/lox^ mice (Figure 3A,B). This was consistent with reduced pathology showing that even temporal deletion of IL‐4Rα has significant impact in TH2 allergic airway response, a phenotype we have mainly observed in global deletion of the gene. We analyzed phosphorylation of downstream transcription factor, signal transducer, and activator of transcription 6 (STAT6) by flow cytometry in lung, mLNs, and lung CD4^+^ T cells (Figure 3C,D). There was a significant reduction in phosphorylated STAT6 expression in TAM‐Rosa^creERT2^IL‐4Rα^−/lox^ mice and IL‐4Rα^−/−^ mice compared to OIL‐Rosa^creERT2^IL‐4Rα^−/lox^ mice.

We then assessed the effect of temporal IL‐4Rα deletion on humoral responses by measuring serum titers of OVA‐specific IgG_1_, IgG_2a_, IgE (Figure 3E) and total IgE (Figure 3F). There was significantly reduced OVA‐specific IgG_1_, IgE, and total IgE in TAM‐Rosa^creERT2^IL‐4Rα^−/lox^ mice when compared to OIL‐Rosa^creERT2^IL‐4Rα^−/lox^ mice, indicating a reduction of type‐2 associated antibody secretion by B cells. These results correlate with a reduction in AHR, mucus secretion, and allergic lung inflammation, when IL‐4Rα was temporally deleted prior to challenge with OVA. Taken together, these results demonstrate that temporally deletion of IL‐4Rα has a profound effect on induction of T_H_2 type allergic airway responses in sensitized mice and in subsequent disease development.

### 3.4 Deletion of IL‐4Rα signaling after established allergic airway disease reduces AHR and airway inflammation

**Figure 4:**
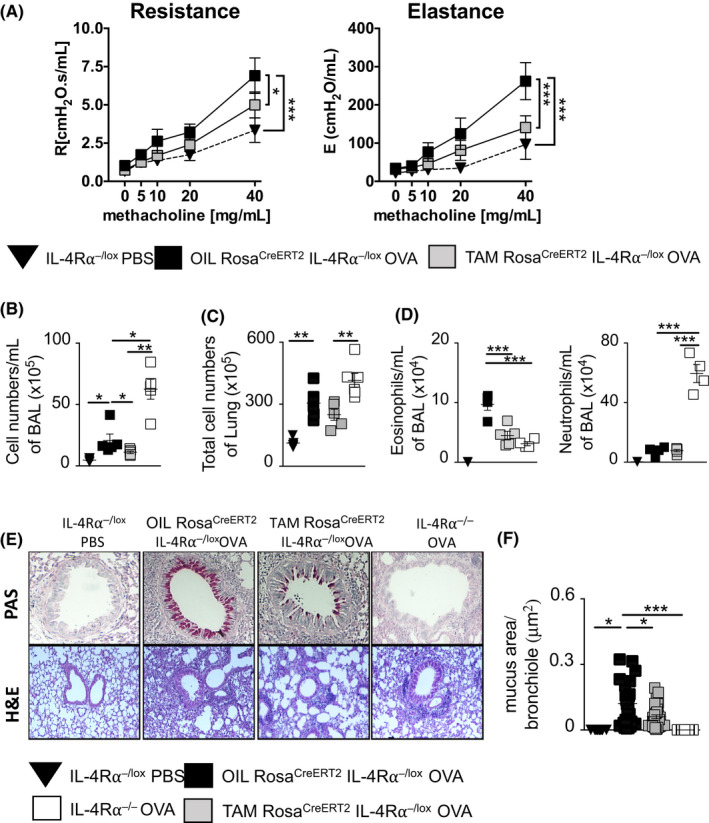
Therapeutic deletion of the IL‐4Rα reduces airway hyperresponsiveness and allergic airway inflammation. Mice (ROSA^cre ERT2^IL‐4Rα^−/lox^) were sensitized with ovalbumin (OVA)/Alum on days 0, 7, and 14 challenged with OVA on days 23‐25, fed (tamoxifen) TAM or OIL on days 27 to 30 by oral gavage, and then challenged with OVA on days 34‐36. Analysis was done on day 37. A, Airway resistance and airway elastance measured with increasing doses of methacholine (0‐40 mg/mL). B, Total bronchoalveolar lavage fluid cells. C, Total lung cells. D, Total number of neutrophils (CD11c^low^CD11b^high^Ly6G^high^) and eosinophils (CD11c^low^ CD11b^high^ Ly6G^low^ SiglecF^hi^) in the BALF were stained and analyzed by flow cytometry. E, Histology analyses of lung sections (magnification ×200), stained with hematoxylin and eosin (H&E) (bottom) and periodic acid‐Schiff (PAS) (top). F, Automated quantification of the area (μm^2^) of mucus staining per analyzed bronchiole epithelial lining was carried out using NIS elements imaging software. Shown is means ± SDs from one representative experiment of 3 independent experiments (n = 5‐8). Significant differences are described as *P < .05, **P < .01, ***P < .001

We assessed the temporal requirement of IL‐4Rα signaling in a therapeutic model as described in Figure 1A. Firstly, we assessed lung function and observed a significant reduction in airway resistance and elastance in TAM‐Rosa^creERT2^IL‐4Rα^−/lox^ compared to OIL‐Rosa^creERT2^IL‐4Rα^−/lox^ littermate controls (Figure 4A). We also observed significantly increased resistance and elastance in OVA‐challenged OIL‐treated Rosa^creERT2^IL‐4Rα^−/lox^ when compared to PBS‐challenged control mice. This demonstrated a protective therapeutic effect of IL‐4Rα temporal deletion in established allergic airway disease.

We then measured total infiltrating cells in BAL fluid and found that they were reduced in TAM‐Rosa^creERT2^IL‐4Rα^−/lox^ mice compared to OIL‐Rosa^creERT2^IL‐4Rα^−/lox^ mice (Figure 4B), although the difference in lung tissue was not significant (Figure 4C). This correlated with a reduction in airway eosinophilia in BAL fluid of TAM‐Rosa^creERT2^IL‐4Rα^−/lox^ mice compared to the OIL‐Rosa^creERT2^IL‐4Rα^−/lox^ mice (Figure 4D). Global IL‐4Rα‐deficient mice had significantly higher neutrophilic infiltration in the BAL fluid when compared to TAM‐Rosa^creERT2^IL‐4Rα^−/lox^ mice, which correlated with increased total cell counts in this group (Figure 4B‐D). When analyzing lung pathology by histology, we observed reduced mucus hypersecretion (Figure 4E,F) and lung inflammation (Figure 4E) in TAM‐Rosa^creERT2^IL‐4Rα^−/lox^ mice compared to OIL‐Rosa^creERT2^IL‐4Rα^−/lox^. These data, together with AHR, suggested that IL‐4Rα signaling is crucial in maintaining allergic airway inflammation in established disease.

### 3.5 T_H_2 immune response and antibody production is maintained after temporal deletion of IL‐4Rα signaling in established allergic airway disease

**Figure 5:**
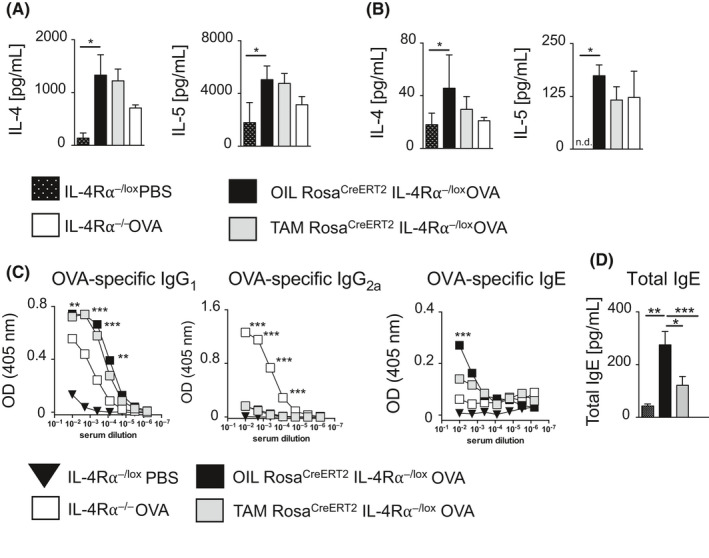
Therapeutic deletion of the IL‐4Rα has a maintained T_H_2 cell–mediated cytokine response even with reduced airway inflammation. T_H_2‐associated cytokine production in therapeutic ovalbumin (OVA) induced allergic asthma was measured in ex vivo restimulated mediastinal lymph nodes (mLNs) by using ELISA. A, anti‐CD3 stimulated and B, OVA stimulated. C, Antibody production in prophylactic depletion model; C, OVA‐specific IgG_1_, IgG_2a,_ and IgE. D, Total IgE. Data show mean ± SDs from 1 representative experiment of 3 independent experiments carried out (n = 5). Significant differences are described as: *P < .05, **P < .01, ***P < .001

We demonstrated that temporal deletion of IL‐4Rα during the effector stage reduced AHR and lung inflammation. We then measured T_H_2 cytokines in mLNs stimulated with anti‐CD3 (Figure 5A) or OVA (Figure 5B). Interestingly, T_H_2 cytokine (IL‐4 and IL‐5) levels were similar between TAM‐Rosa^creERT2^IL‐4Rα^−/lox^ and OIL‐Rosa^creERT2^IL‐4Rα^−/lox^ mice in both anti‐CD3 (Figure 5A) and OVA‐stimulated (Figure 5B) mLNs. We then assessed humoral responses by measuring levels of OVA‐specific antibodies and total IgE. We observed no reduction in OVA‐specific IgG_1_ and IgG_2a_ (Figure 5C), although a significant reduction in total IgE was observed (Figure 5D) along with a slight reduction in OVA‐specific IgE (Figure 5C) when comparing TAM‐Rosa^creERT2^IL‐4Rα^−/lox^ mice and OIL‐Rosa^creERT2^IL‐4Rα^−/lox^ mice. Taken together, these data suggested that the reduced signs of allergic airway inflammation and AHR during temporal deletion of IL‐4Rα were unlikely to be caused by reduced type‐2 cytokines.

**Figure 6:**
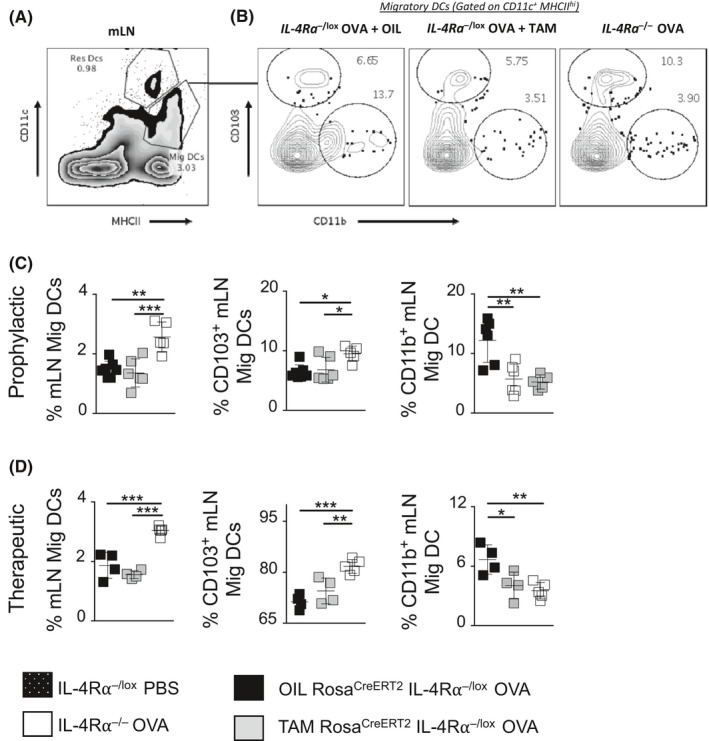
The dendritic cell compartment was altered with temporal depletion of the IL‐4Rα gene. A, A representative gating strategy for migratory dendritic cells (DCs) (CD11c^+^MHCII^hi^) and residential DCs (CD11c^+^MHCII^int^) in mLNs stained by flow cytometry. B, gating strategy of the CD103^+^ migratory DCs and CD11b^+^ migratory DCs. C, Plotted mean ± SDs proportions of migrated cell populations following ovalbumin (OVA) airway exposure in a prophylactic model. D, Plotted mean ± SDs proportions of migrated cell populations following OVA airway exposure in a therapeutic model. Significant differences among groups are represented as (*P < .05, **P < .01, ***P < .001)

Functionally distinct DCs are central in not only inducing, but also suppressing T_H_2 or T_H_17 immune response through secretion of T_H_ cell–polarizing cytokines.^34^, ^35^, ^36^, ^37^, ^38^ We investigated the DC compartment in lung draining LNs (Figure 6A,B). We observed a reduction in migratory CD11b^+^ DCs in both prophylactic (Figure 6C) and therapeutic (Figure 6D) IL‐4Rα temporarily deleted TAM‐Rosa^creERT2^IL‐4Rα^−/lox^ mice compared to the OIL‐Rosa^creERT2^IL‐4Rα^−/lox^ mice. These migratory DCs are implicated in trafficking of antigens to the lymphoid tissue during sensitization and induction of a T_H_2 cell–mediated response.^38^ These results suggest a possible necessity of IL‐4Rα signaling in trafficking of CD11b^+^ DCs for establishing T_H_2 cell–mediated inflammation in allergic airway disease.

### 3.6 T_H_17‐induced neutrophilia is dependent on IL‐4Rα deletion prior to sensitization

**Figure 7:**
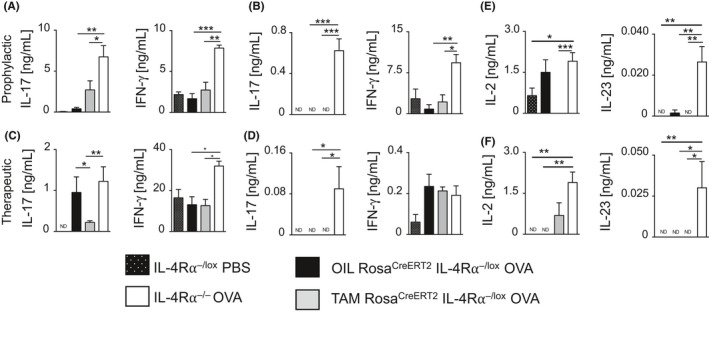
Prophylactic and therapeutic deletion of IL‐4Rα reduces a T_H_17‐associated cytokine response. T_H_1‐ and T_H_17‐associated cytokine production levels were measured in ex vivo restimulated mediastinal lymph nodes (mLNs) by using ELISA in both models. A, Prophylactic anti‐CD3 stimulated and B, ovalbumin (OVA) stimulated cytokine (IL‐17 and IFN‐γ) production. C, Therapeutic anti‐CD3 stimulated and D, OVA‐stimulated cytokine (IL‐17 and IFN‐γ) production. E, OVA stimulated cytokine (IL‐2 and IL‐23) production in prophylactic model and F, in therapeutic model. Data show mean ± SDs from 1 representative experiment of 3 independent experiments. Significant differences are shown as: *P < .05, **P < .01, ***P < .001

We observed airway neutrophilia in IL‐4Rα^−/−^ mice, but not in TAM‐Rosa^creERT2^IL‐4Rα^−/lox^ mice in both prophylactic and therapeutic deletion of IL‐4Rα signaling (Figures 2D and 4D). We showed previously that increased production of IL‐17, after disruption of IL‐4Rα expression on CD4^+^ T cells, was responsible for increased IL‐17 production and airway neutrophilia.^11^ IL‐17 levels were measured in supernatants of ex vivo anti‐CD3 (Figure 7A,C) and OVA restimulated mLNs (Figure 7B,D) in both prophylactic (Figure 7A,B) and therapeutic models (Figure 7C,D). IL‐17 was found to be increased in IL‐4Rα^−/−^ mice, but not in TAM‐Rosa^creERT2^IL‐4Rα^−/lox^ mice (Figure 7A‐D). Additionally, IFN‐γ was increased significantly in IL‐4Rα^−/−^ mice compared to the other groups in the prophylactic model and anti‐CD3 stimulated mLNs of prophylactic model mice (Figure 7A‐D), further confirming that the anti‐T_H_2 effect observed in the TAM‐Rosa^creERT2^IL‐4Rα^−/lox^ was IFN‐γ‐independent. However, IFN‐γ release was similar in mLNs stimulated with OVA of the IL‐4Rα^−/−^ and TAM‐Rosa^creERT2^IL‐4Rα^−/lox^ mice (Figure 7D). We further investigated a possible DC compartment responsible for the consequential T_H_17 response (Figure 6A and 6). There was increased migratory CD103^+^ DCs in mLN of IL‐4Rα^−/−^ mice compared to TAM‐Rosa^creERT2^IL‐4Rα^−/lox^ and OIL‐Rosa^creERT2^IL‐4Rα^−/lox^ mice in both prophylactic (Figure 6C) and therapeutic (Figure 6D) models. CD103^+^ DCs have been shown to control T_H_17 polarization by secreting polarizing cytokines IL‐23 and IL‐2.^38^, ^39^ We also found increased levels of IL‐23 and IL‐2 in OVA‐restimulated mLNs in IL‐4Rα^−/−^ mice compared to TAM‐Rosa^creERT2^IL‐4Rα^−/lox^ mice in the prophylactic model (Figure 7E). In a therapeutic model, IL‐4Rα^−/−^ mice showed an increase in IL‐2 and IL‐23 which was significant compared to the other groups, except for TAM‐treated mice (Figure 7F). We further validated our findings by temporal deletion of IL‐4Rα before sensitization (Figure S2A). We observed increased BAL fluid neutrophilia, mLN CD103^+^ migratory DCs, IL‐17, and IL‐23 in TAM‐Rosa^creERT2^IL‐4Rα^−/lox^ mice when compared to OIL‐Rosa^creERT2^IL‐4Rα^−/lox^ mice (Figure S2B‐E), a phenotype similar to what we observe in a global knockout mice. These results highlight a potential involvement of migratory CD103^+^ DCs together with IL‐23 in maintaining expansion of T_H_17 cells observed in IL‐4Rα deletion prior to sensitization. Our approach of temporal IL‐4Rα chain deletion supports a critical role in establishment and maintenance of disease and further highlights its therapeutic potential in many asthma disease endotypes.

## 4 DISCUSSION

The critical importance of IL‐4/IL‐13 axis as a driver of T_H_2 immunity in allergic asthma has been established with IL‐4Rα being central mediator of disease pathology.^1^, ^4^ We and others have previously demonstrated cell‐specific function of IL‐4Rα signaling in the development of allergic disease.^1^, ^11^, ^16^, ^18^, ^19^, ^20^, ^21^, ^40^ However, the temporal role of IL‐4Rα signaling in vivo, during sensitization and effector phases of allergic disease, is not completely clear. In this study, we developed an inducible IL‐4Rα deletion model, allowing us to conditionally delete receptor signaling. Using this approach, we were able to investigate the role of IL‐4/IL‐13 signaling during onset and effector phase of OVA‐induced allergic airway disease in systemically sensitized animals. Our findings show that temporal deletion of IL‐4Rα signaling postsensitization prevents development of OVA‐induced AHR, eosinophilia, and goblet cell hyperplasia (summarized in Table S1). Hence, abrogation of IL‐4Rα signaling should have significant therapeutic benefit for T_H_2‐type allergic asthma. Our study contributes to already existing literature on the central role of IL‐4Rα subunit in allergic asthma and may guide currently ongoing phase III clinical trials targeting IL‐4Rα in various asthma endotypes.

IL‐4Rα signaling is important in establishing a T_H_2‐driven allergic airway pathology, and its absence, for example, IL‐4Rα‐deficient mice, leads to amelioration of IL‐4/IL‐13‐dependent allergic airway disease.^41^, ^42^, ^43^ We observed a reduction in AHR, eosinophilia, mucus hypersecretion, and pathology during temporal deletion of IL‐4Rα after sensitization phase or during effector phase. This indicates that anti‐IL‐4Rα therapies may be suitable for therapeutic treatment of acute allergic disease patients and possibly prophylactic intervention in asymptomatic, but sensitized patients, who represent 50%‐60% of asthma patients^44^, ^45^ and are highly at risk of developing asthma or eczema.^46^, ^47^

We further showed that the diminished T_H_2 cell response in our study is not caused by an increased IFN‐*γ* response as previously suggested.^48^ The reduction in T_H_2 responses in our study correlated with reduced STAT6 phosphorylation.^43^ Of significant note is the potential requirement for IL‐4Rα signaling in migration of CD11b^+^ DCs to secondary lymphoid tissues for antigen sensitization and priming of a T_H_2 cell–mediated immune response. This would be consistent with previous studies showing ILC2‐derived IL‐13 being important in priming migration of DC to mediastinal lymph nodes through CCL21 chemoattractant.^49^ It is also possible that IL‐4Rα in DC is crucial for recruitment of memory T_H_2 cells to the lung through production of chemokines such as CCL17.^38^, ^50^ IL‐4 and IL‐13 have been shown to regulate chemokine production by DCs or macrophages^51^, ^52^ and monocyte‐derived DCs have been implicated in being predominant chemokine producers, which are known to regulate migration of T_H_2 cells.^38^ This modulation of the DC compartment suggests an additional diverse mechanism involved in augmenting allergic airway inflammation with the temporal deletion of IL‐4Rα signaling postallergen sensitization.

Deletion of the IL‐4Rα subunit after established T_H_2 disease shows a reduction in OVA‐induced AHR and airway inflammation despite a persistent T_H_2 cytokine production. This is suggestive of a maintained T_H_2 memory cell recall from systemic OVA sensitization which is IL‐4/IL‐13 independent.^53^, ^54^, ^55^, ^56^ This is in contrast to a similar temporal deletion of IL‐4Rα signaling prior to secondary infection with *Nippostrongylus brasiliensis* showing a recall role in driving type‐2 immune responses and clearance of the parasites.^33^ In this helminth infection model, IL‐4Rα signaling is vital in not only initiating, but also maintaining a functional T_H_2‐driven pathology and its associated cytokines. However, we show evidence that IL‐4Rα signaling is not necessary for restimulation of a new T_H_2 cell response in allergic airway disease. This is consistent with previous studies where blocking IL‐4/IL‐13 axis after an established T_H_2 response with an antagonist did not affect ongoing cytokine production by IL‐4‐secreting CD4^+^ T cells.^56^ Recently, using single‐cell RNA sequencing, it was shown that basal stem cells from nasal epithelial polyps have a strong IL‐4/IL‐13 memory signature that persists even after dupilumab treatment.^57^ This stem cell memory may be what drives disease persistence especially in established T_H_2 airway responses.

The successful utility of targeting IL‐4Rα signaling has led to clinical trials for dupilumab, a human monoclonal antibody for anti‐T_H_2 therapy in allergic asthma patients and atopic dermatitis.^12^, ^13^, ^58^ Concern arises on unprecedented T_H_1/T_H_17 polarizing inflammation with anti‐T_H_2 interventions in humans and mice.^4^, ^24^, ^25^, ^27^, ^59^, ^60^ Our mouse model clears a T_H_2 response without inducing any potential T_H_1/T_H_17 responses even with established disease, thus emphasizing on a potential benefit of anti‐IL‐4Rα therapies as a viable T_H_2 treatment option. Conversely, Stat6‐deficient or IL‐4Rα‐deficient mice have shown increased IgG_2a_ and IgG_2b_ secretion even with resolution of T_H_2 inflammation.^4^, ^59^ Recently, Rorγt‐deficient T cells were shown to suppress T_H_2 and T_H_17 differentiation via upregulated BCL6 expression in the airways.^61^ Bcl6, a transcriptional repressor which antagonizes both Rorγt and Gata3,^62^, ^63^ is high in IL‐4‐committed T follicular helper (Tfh) cells, a precursor of T_H_2 cells upon secondary antigen challenge.^64^ The anti‐T_H_2 outcome observed with Rorγt deficiency in T cells 61 could be a result of impaired Tfh developmental plasticity to develop into effector T cells.^64^ It is likely that temporal deletion of IL‐4Rα on T cells might disrupt Tfh cell responses during sensitization phase, preventing their IL‐4 commitment into effector T_H_2 cells.^63^

Development of T_H_17 responses has been suggested as a potential detrimental side‐effect of anti‐allergic therapies targeting the IL‐4/IL‐13/IL‐4Rα signaling pathway.^65^ The exact mechanism of how the T_H_17 response is potentiated is elusive. In our previous work, we have shown that disruption of IL‐4Rα‐signaling specifically on CD4^+^ T cells results in antigen‐specific T_H_17 responses after allergic sensitization.^11^ Similar to the global IL‐4Rα knockout mice, deficiency of IL‐4Rα subunit on CD4^+^ T cells was prior to sensitization. In this study, T_H_17 responses were only observed in global IL‐4Rα‐deficient mice, but not in TAM‐Rosa^creERT2^IL‐4Rα^−/lox^ mice, where IL‐4Rα signaling was disrupted only after sensitization. Additionally, with the observed long‐lasting effects of embryonic deletion of IL‐4Rα and that of “conditional” knockout of IL‐4Rα at presensitized period, the protective effect against challenge with allergen could be due to the protective effect against IL‐13 because of the deleted IL‐4Rα signaling. This is further seen in the therapeutic model where in the presence of a residual IL‐13 production, the protective effect against IL‐13 still remains with an observed diminished airway hyperresponsiveness. Thus, our study supports the concept that IL‐4Rα signaling directly prevents differentiation of CD4^+^ T cells into T_H_17 cells during allergen sensitization and reveals a temporal IL‐4Rα dependency in development of a T_H_17 airway inflammation. Strikingly, with the absence of IL‐4Rα signaling prior to sensitization, we observed a modulation of a DC compartment in lymphoid tissue. A predominant increase migratory CD103^+^ DC profile and antigen‐specific IL‐23 secretion were observed and are both known to be responsible for maintenance of memory T_H_17 cells.^66^ Shalaby et al similarly revealed pathogenic T_H_17 cells in airway inflammation which was mainly driven by cDC IL‐23. However, their model relied on environmental adjuvants (HDM and LPS) driving a TLR4‐dependent co‐stimulation with OVA to expand or maintain chronic airway inflammation.^39^

In conclusion, we provide further evidence for a therapeutic potential for blocking IL‐4Rα signaling in acute disease cases as well as prophylactic possibility in cases of asymptomatic atopic patients in T_H_2‐type allergic airway inflammatory responses.

## CONFLICTS OF INTEREST

The authors declare that they have no conflicts of interest.

## AUTHOR CONTRIBUTIONS

FB, FK, and SH conceived and supervised study. JK, SH, FK, and MS performed the experiments. JK, SH, FK, and FB analyzed the data. JK, SH, FK, and FB wrote the paper. All authors discussed the results and commented on the manuscript.
