# Supplementary material for: Therapeutic and prophylactic deletion of IL‐4Ra‐signaling ameliorates established ovalbumin induced allergic asthma
Source: Allergy. 2020 Jan 30;75(6):1347–60. doi: 10.1111/all.14137 (PMC7318634; doi:10.1111/all.14137)
Supplement: Supplementary file 1 [file ALL-75-1347-s001.pdf]

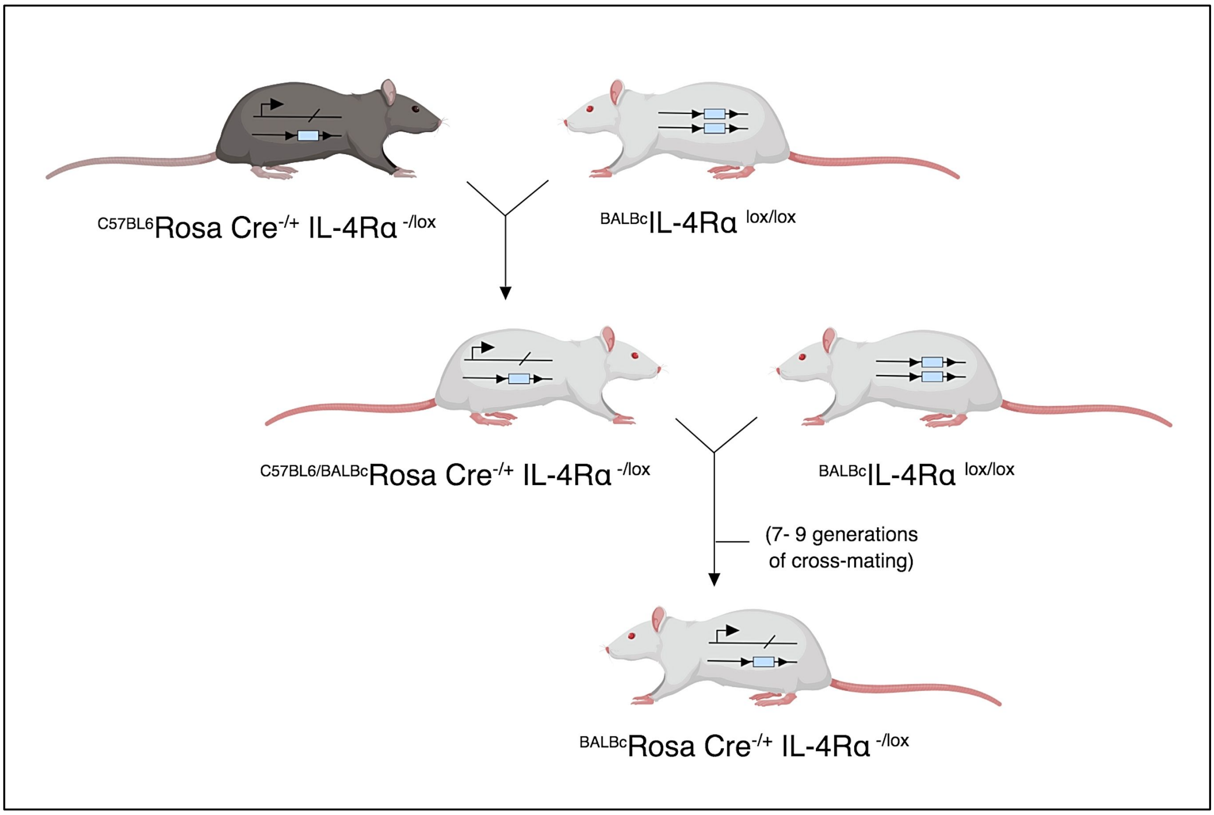

Supplementary Figure 1. Mouse breeding schematic of Rosa$^{Cre-/+}$ IL-4Rα$^{-/lox}$ BALB/c mice

Rosa$^{Cre-/+}$ IL-4Rα$^{-/lox}$ C57BL/6 mice were intercrossed with IL-4Rα$^{Lox/Lox}$ BALB/c mice to generate Rosa$^{Cre-/+}$ IL-4Rα$^{-/lox}$ BALB/c mice.
