# Supplementary figures and images for: Therapeutic and prophylactic deletion of IL‐4Ra‐signaling ameliorates established ovalbumin induced allergic asthma
Source: Allergy. 2020 Jan 30;75(6):1347–60. doi: 10.1111/all.14137 (PMC7318634; doi:10.1111/all.14137)

## Slide 1
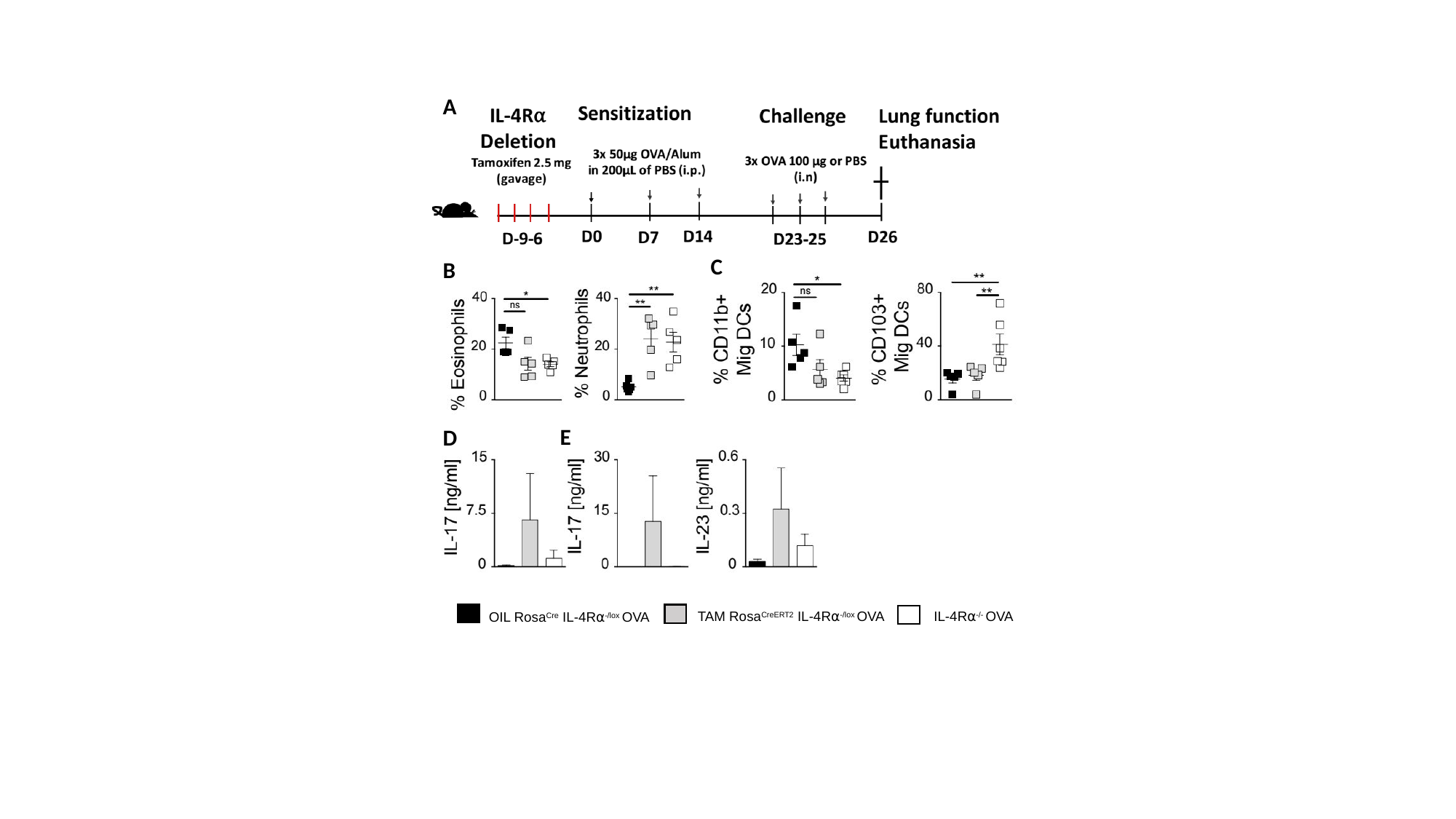

A
C
B
E
D
TAM RosaCreERT2 IL-4R⍺-/lox OVA
IL-4R⍺-/- OVA
OIL RosaCre IL-4R⍺-/lox OVA

Supplement: Supplementary file 2 [file ALL-75-1347-s002.pptx]
