# Supplementary material for: Therapeutic and prophylactic deletion of IL‐4Ra‐signaling ameliorates established ovalbumin induced allergic asthma
Source: Allergy. 2020 Jan 30;75(6):1347–60. doi: 10.1111/all.14137 (PMC7318634; doi:10.1111/all.14137)
Supplement: Supplementary file 3 [file ALL-75-1347-s003.docx]

**Supplementary material**

**Full title** Therapeutic and prophylactic deletion of IL-4Rα-signaling ameliorates established ovalbumin induced allergic asthma

**Authors’ list**:

Jermaine Khumalo, Frank Kirstein, Martyna Scibiorek, Sabelo Hadebe, Frank Brombacher


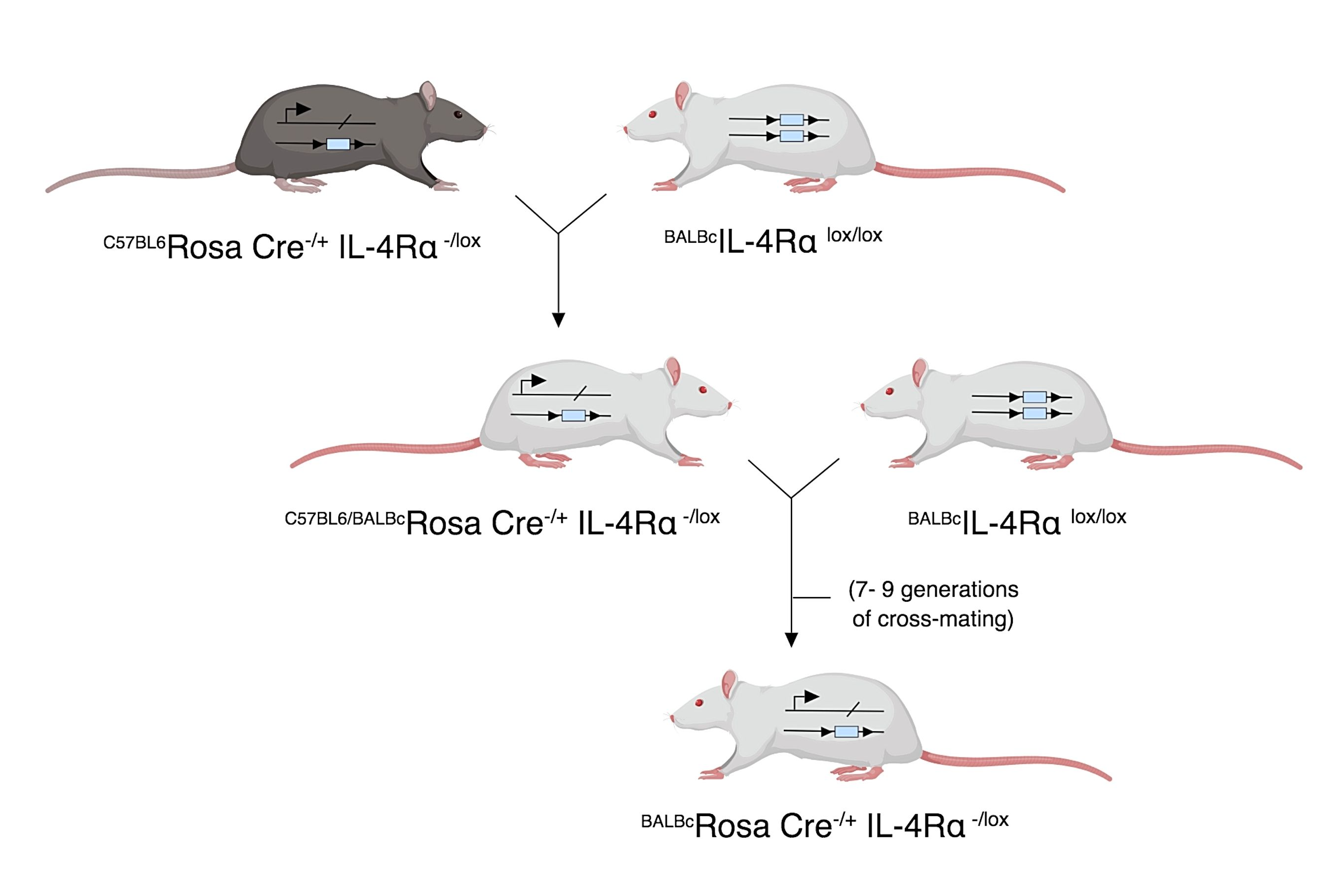

Table S1: Comparison of temporal genetic deletion of IL-4Rα gene vs embryonic deletion

|  | Embryonic deletion | Prophylactic | Therapeutic | Pre-sensitisation |
| --- | --- | --- | --- | --- |
| Humoral response | IgE low/IgG2a high | IgE low/IgG2alow | IgE low/IgG2alow | IgE low/IgG2alow |
| Eosinophils | + | + | + | + |
| Neutrophils | +++ | + | + | +++ |
| T_H_2 cytokines | + | + | +++ | + |
| T_H_17 cytokines | +++ | + | + | +++ |

+++ high, ++ intermediate, + Low

**Supplemenatry Figure legends**

**Supplementary Figure 1. Mouse breeding schematic of Rosa^Cre-/+^ IL-4Rα^-/lox^ BALB/c mice**

Rosa^Cre-/+^ IL-4Rα^-/lox^ C57BL/6 mice were intercrossed with IL-4Rα^Lox/Lox^ BALB/c mice to generate Rosa^Cre-/+^ IL-4Rα^-/lox^ BALB/c mice.

**Supplementary Figure 2:** **Pre-sensitisation deletion of IL-4Rα induces T_H_17-associated cytokine response and neutrophilia.**

**A**, Schematic diagram of IL-4Rα deletion using the TAM inducible mouse model (ROSA^creERT2^IL-4Rα^-/lox^ mice) before sensitisation. Mice were fed tamoxifen (TAM) (2.5 mg) orally for 4 days and then sensitised with ovalbumin (OVA)/Alum on days 0, 7 and 14, challenged with OVA on days 23-25 and analyses done on day 26.

**B,** Percentages of eosinophils (CD11c^low^ CD11b^high^ Ly6G^low^ SiglecF^hi^) and neutrophils (CD11c^low^CD11b^high^Ly6G^high^) in the bronchioalveolar lavage fluid (BALF) were stained and analysed by Flow cytometry.

T_H_17 associated cytokine production in OVA induced allergic asthma were measured in *ex vivo* re-stimulated mediastinal lymph node (mLN) by ELISA.

**C,** Percentages of migrated cell populations following OVA airway, gating strategy is as shown in Figure 6.

**D**, anti-CD3 stimulated and

**E**, OVA stimulated.

Data shows mean ±SDs from 1 experiment. Significant differences are shown as: *p < .05, **p < .01. ns, not significant.
